# Evaluation of Surface Roughness and Defect Formation after The Machining of Sintered Aluminum Alloy AlSi10Mg

**DOI:** 10.3390/ma13071662

**Published:** 2020-04-03

**Authors:** Grzegorz Struzikiewicz, Andrzej Sioma

**Affiliations:** 1 Production Engineering Institute, Mechanical Faculty, Cracow University of Technology, 31-155 Kraków, Poland; 2 Department of Process Control, Faculty of Mechanical Engineering and Robotics, AGH University of Science and Technology, 30-059 Kraków, Poland; andrzej.sioma@agh.edu.pl

**Keywords:** machining, sintered aluminum, 3D surface roughness parameters, surface defects

## Abstract

This article presents selected issues related to the workpiece surface quality after machining by the laser sintering of AlSi10MG alloy powder. The surfaces of the workpiece were prepared and machined by longitudinal turning with tools made of sintered carbides. The occurrence of breaches on the machined material surface was found, which negatively influence the values of 3D surface roughness parameters. The occurring phenomena were analyzed and proposals for their explanation were made. Guidelines for the machining of workpieces achieved by the laser sintering of powders were developed. The lowest value of the 3D roughness parameters was obtained for *f* = 0.06 mm/rev, *a_p_* = 0.5–1.0 mm, and for the nose radius of cutting insert *r_ε_* = 0.8 mm. The results of research on the effect of cutting parameters on the values of parameters describing the surface quality are presented. Topography measurements and 3D surface roughness parameters are presented, as well as the results of a microscopic 3D surface analysis. Taguchi’s method was used in the research methodology.

## 1. Introduction

The aim of currently observed directions and development trends visible in manufacturing techniques is to meet the requirements for dimension and shape accuracy and surface quality. One of the solutions used is a hybrid machining that combines subtractive manufacturing with additive machining. This approach is in accordance with the “All-in-One” and “Done-in-One” manufacturing philosophy used in multi-purpose numerically controlled machine tools produces by, e.g., DMG and Mazak. In this case, the hybrid manufacturing consists of the printing (additive manufacturing) of a workpiece or its piece, and then carrying out the final machining in the form of the burr removal (subtractive machining) of selected surfaces in order to ensure the required accuracy and quality level. The combination of subtractive machining with AM (Additive Manufacturing) is also beneficial in terms of manufacturing costs of machine parts [1]. This mainly concerns the manufacturing of large-size workpieces with thin-walled elements.

Both technologies have a number of advantages and disadvantages. In the case of AM, the advantage is the capability to achieve the workpiece of "any shape", while the disadvantage is still a lower surface quality and dimension and shape accuracy compared to workpieces made by the subtractive machining [2,3,4]. In addition, DMLS/SLM (Direct Metal Laser Sintering/Selective Laser Melting) technology allows for the manufacturing of complex and minute spatial structures, which cannot be achieved with casting and subtractive methods. Since the vast majority of manufactured workpieces is made of metal, the industry interest focuses on AM technology in terms of the production of fully functional metal workpieces.

Methods of additive manufacturing machine parts using metal alloy powders are innovative and still under research. They demonstrate a number of new problems requiring scientific and technological development. The most commonly used 3D printing technologies are FDM (Fused Deposition Modeling), SLS (Selective Laser Sintering), SLA (Stereolithergaphy), and others [1]. SLM technology, on the other hand, is a selective laser sintering and remelting of powdered metals, which are applied layer by layer until a fully durable workpiece is ready [5]. The aim of currently conducted research is to replace the casting technology with SLM and DMLS [2].

Metal materials obtained with the additive technology feature porosity and areas of varied material cohesion. AlSi10Mg has a good strength, corrosion resistance, low density, and high thermal conductivity compared with other alloys and is of tenfound in aerospace and automotive interior AM components, and in functional prototypes [2,6,7]. 

The surface quality obtained by DMLS/SLM is similar to that of castings by the lost model, with a surface roughness of *Ra* = 4–20 µm, depending on the alloy used and layer thickness. The characteristic property of workpieces manufactured with AM technology is the layered structure of the material. DMLS/SLM technologies most often use the ytterbium fiber laser operating in the infrared band (formerly weaker CO_2_ lasers were used). The workpieces are manufactured by applying thin layers of metal powder (0.01–0.08 mm thickness). The process of material application usually includes the levelling out of the remelting roughness from the previously applied layer [2,8]. Recent publications on the additive manufacturing have mainly concerned the issues of surface metrology performed after additive manufacturing. As shown by Townsend et al. [9], in industrial conditions, the most commonly used parameters for surface quality assessment are 2D parameters describing the surface roughness (e.g., *Ra*, *Rz* etc.). As the material obtained by the additive method is porous and has a layered structure, the evaluation of the surface based on the above-mentioned parameters may not be insufficient [5,10]. Triantaphyllou et al. [11] presented the comprehensive literature analysis on surface texture metrology for metal additive manufacturing. They have shown that texture characterization is mainly based on the measurements of surface profiles and 2D parameters, of which *Ra* is the most commonly used. 

However, the three-dimensional topography of a surface analysis is becoming more and more popular. In turn, Gao et al. [12] showed that there are still neither rules nor guidelines for additive manufacturing and indicated the need to standardize it. Based on the analysis of the surface measurement methods of workpieces made with SLM, Diatlov et al. [13] presented the concept of a roughness spectrum as an alternative to roughness value *Ra*. With reference to SLM, Rao et al. [10] demonstrated that, using optimal laser parameters, this technology can be implemented to manufacture workpieces made of A357 aluminum alloy, with the density and mechanical properties of the cast alloy standard and of a low porosity. However, different laser parameters also caused different melt pool size and morphology after SLM. Similar studies were conducted by Calignano et al. [14], who investigated the influence of process parameters, such as scan speed, laser power, and hatching distance (the perpendicular distance between successive laser scan lines), on the surface finish of direct metal laser sintered AlSi10Mg surfaces. Analyses of these issues available in the literature mainly concern the surface characteristics of workpieces obtained with additive technologies [1] rather than the subtractive machining. The authors of the published analyses focus mainly on the search for relationships between adjustable parameters of additive technology and parameters describing the surface quality, especially surface roughness. For example, Read et al. [7] described the influence of process parameters on the porosity of SLM-manufactured workpieces. He demonstrated that such surfaces have cracks and show the presence of significant amounts of unmelted powder, which results in the growth of material cracks. In turn, Strano et al. [15] analyzed the workpiece made of 316L steel alloy and carried out surface roughness tests. The analysis demonstrated an increasing density of spare particles positioned along the step edges, as the surface sloping angle increases. When the layer thickness is comparable to the diameter of the particle, the particles attached along the step edges can fill the gaps between the successive layers, thus affecting the actual surface roughness. Mumtaz and Hopkinson [16] carried out similar analyses, but for SLM of Inconel 625 alloy. They analyzed the processing parameters that simultaneously influence the roughness of the top and side surfaces of the manufactured workpiece. This demonstrated that higher peak powers tended to reduce top surface roughness and reduce side roughness as recoil pressures flatten out the melt pool and reduce balling formation by increasing the wettability of the melt. The increased repetition rate and reduced scan speed reduced top surface roughness but increased side roughness. Moreover, the authors (Grimm et al. [17]) analyze the correlation between the surface orientation of SLM parameters and *Sdr* (developed interfacial area ratio). In turn, [16] found that *Sa* and *Sq* were suitable measurement parameters for SLM. 

The surface condition depends on the method and conditions of the workpiece material manufacturing (casting, forging, additive methods) and the methods and conditions of machining. Guo et al. [18] presents a theoretical and experimental investigation on the ultra-precision machining of V-groove structures on rapidly solidified aluminum RSA-905 using single-crystal diamond tools. The authors analyzed chip flow and material removal phenomena and effects of depth of cut and feed rate on cutting forces, surface quality, and form accuracy. The results showed that 15 nm Ra surface roughness were obtained on V-groove surface under the best machining condition. Guo et al. [19] carried out similar analyses on the surface integrity of rapidly solidified aluminum by magnetic field-assisted finishing. The effect of abrasive and polishing speed conditions on material removal and surface roughness was investigated. The results show that a low surface roughness was obtained under conditions using the SiC abrasive with a grit size of 12 μm at a polishing speed of 400 rpm or using the Al2O3 abrasive with a grit size of 5 μm at a polishing speed of 800 rpm. Therefore, there is a need to analyze the application of cutting the metal workpiece made by additive technology, and the impact of the subtractive machining (i.e., machining parameters such as feed, cutting speed, and cutting depth) on the surface quality of thus produced workpieces [3].

The authors of [20] compared the effect of aluminum alloy’s cutting parameters in cast and sintered forms on values of the cutting force components and dimensional and shape accuracy. They showed that the burrs created during the passage of the cutting tool influence the dimensional and shape accuracy. Matras [21], on the other hand, analyzed the milling process of sintered aluminum and optimization of *Ra* and *Rz* surface roughness parameters. However, most of the research results described in the literature concern the machining of cast materials. Cutting forces in aluminum alloys are usually low and result from their lower mechanical strength [2]. The surface roughness during the subtractive machining of aluminum is significantly influenced by the hardness of the alloy and microstructural properties [22]. While machining alloys of a higher hardness, the values of the parameters describing surface roughness usually decrease [2,22], as hardness limits the adhesion of the material to the cutting edge of the tool. However, BUE (Build Up Edge) formation and the random extraction of hard particles from the material may occur. The high chemical affinity of aluminum alloys to cutting tool coating materials, such as TiC or Al_2_O_3_, causes the machined material to accumulate on the tool surface. This leads to deterioration of the material surface roughness due to the continuous adhesion of particles to the workpiece surface [2,22]. On the basis of the relationships obtained from the research, mathematical models are also developed to determine the values of selected surface topography parameters (Jayaraman et al. [23] Pawlus et al. [24] or Singh et al. [25]).

An important issue, also concerning aluminum alloys, is the optimization of the cutting process due to chip form and the choice of cutting parameters. The relationship between the chip form and cutting conditions was presented by Słodki et al. [4]. The authors presented investigations related to the effectiveness of selected chip breakers working in the local machining environment. Recommendations for cutting condition correction for the purpose of achieving an acceptable chip form were presented.

Currently, there are no dedicated procedures to optimize the cutting of workpieces produced by additive manufacturing. The authors attempted to establish a procedure for finding optimal cutting parameters for the finishing machining of laser sintered AlSi10Mg alloy, taking into account the criterion of the machined surface quality described by 3D surface roughness parameters. The analysis of the longitudinal turning of the cylinder made of sintered aluminum AlSi10Mg and the roughness, topography, and microscopic measurements of the machined surface were carried out.

## 2. Materials and Methods 

In order to carry out the tests, a sample was prepared for testing by the selective sintering and remelting of powdered aluminum with a laser. The part was obtained using Renishaw’s (Wotton-under-Edge, New Mills, UK) AM 250 with additive technology by selective laser sintering of AlSi10Mg aluminum powder. The properties of AlSi10Mg aluminum powder are presented in Table 1. The mechanical properties of the material and its chemical composition are presented in Table 2 and Table 3. This alloy is used for large castings of complex shape and high strength, heavily and medium loaded, among others, in gearbox housings, steering gear housings, and blocks of internal combustion engines in motor vehicles.

**Figure 1:**
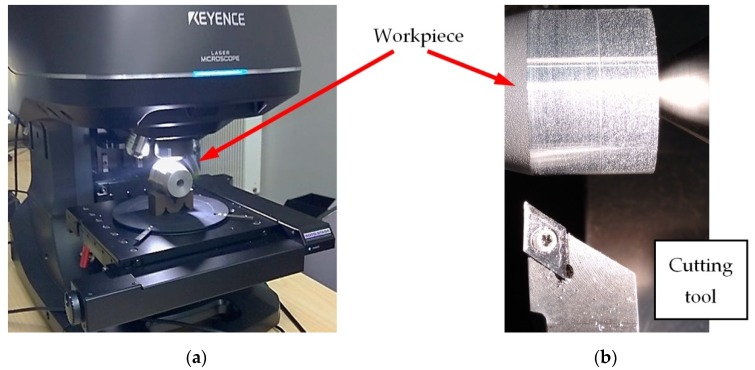
Measuring station (**a**) for surface imaging with the Keyence VK-X1000 microscope and (**b**) the experimental station.

The following measurements were taken: surface roughness and topography and the microscopic measurements of the machined surface. The measurements were captured with Talysurf 50 surface profiler manufactured by Taylor Hobson (2 New Star Rd, Leicester, UK). The microscopic analysis of the machined surface was carried out using a VK-X1000 3D microscope by Keyence (Osaka, Japan) (Figure 1) with a resolution of 0.5 nanometers in the Z axis and 130 nanometers in the XY axis. The imaging field was 705 microns in the X axis and 528 microns in the Y axis.

The analysis of the influence of cutting parameters on the surface roughness and dimension and shape accuracy of machined parts is often carried out on the basis of various methods, such as Taguchi [26,27] or its modifications [23,28]. The authors of the paper [3] presented the analysis of various optimization techniques used in manufacturing processes. The experimental research plan was developed according to the Taguchi method. The influence of variable cutting parameters, i.e., the feed rate, the speed and depth of cutting (*f*, *v_c_*, *a_p_*), and the nose radius of the cutting insert *r_ε_*_,_ on the values of the 3D surface roughness parameters was analyzed. In the statistical analysis of the test results, the model of the matching function according to Equation (1) was adopted.

(1) $$Y_{1}=y-\varepsilon =b_{0}x_{0}+b_{1}x_{1}+b_{2}x_{2}+b_{3}x_{3}+b_{4}x_{4},$$

where:

—*Y*_1_ is the estimated response based on first order equation;

—*y* is the measured parameter (e.g., roughness parameter) on a logarithmic scale;

—*x*_0_ = 1 dummy variable; 

—*x*_1_−*x*_4_ are the logarithmic transformations of cutting speed and the feed and depth of cut;

—ε is the experimental error;

—*b* values are the estimates of corresponding parameters.

The S/N (signal-to-noise) ratio analysis strategy was adopted as “the lowest-the best” according to Equation (2):

(2) $$\frac{S}{N}=-10\cdot log\left(\frac{1}{n}\overset{n}{\underset{i=1}{\sum }}y_{i}^{2}\right)$$

where *y_i_* is the respective characteristic and *n* is the number of observations.

In the cutting tests, DCGT 11T304-AS (*r_ε_* = 0.2 mm) and DCGT 11T308-AS (*r_ε_* = 0.8 mm) cutting inserts of type IC20 by ISCAR (Tefen, Israel) were used. The adopted ranges of the cutting parameter values are: *f* = 0.06; 0.12; 0.17; 0.25 mm/rev, *a_p_* = 0.5; 1.0 mm and *v_c_* = 200; 300 m/min. The values of the cutting parameters are within the range of cutting parameters recommended by the tool manufacturer.

Table 4 presents the test plan together with the actual values of the cutting parameters used in research.

## 3. Results and Discussion

In accordance with the adopted test plan, tests were carried out on the cutting (i.e., longitudinal turning) of the workpiece made by laser sintering. The microscopic observations and measurements of the selected 3D parameters of the surface roughness were performed afterwards.

**Figure 2:**
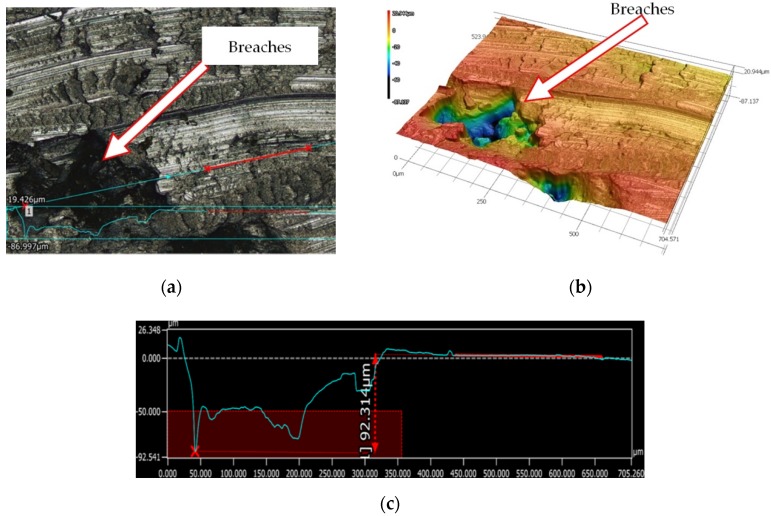
Example of surface measurement with a 3D microscope: (**a**) 2D surface view, (**b**) turned surface imaging (3D presentation), and (**c**) material breach measurement on a machined surface.

**Figure 3:**
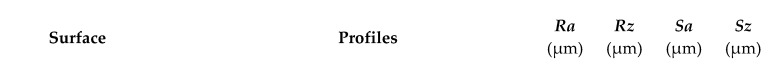

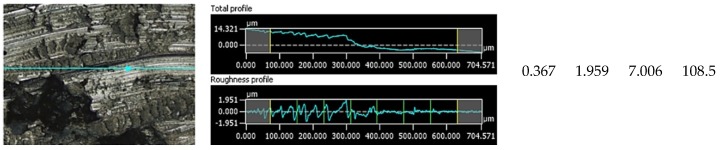
Example of measurement of 2 and 3D parameters of surface roughness with the Keyence microscope.

The analysis of the microscopic measurement results show numerous breaches occurring after the machining of the surface of the workpiece made with additive technology. Figure 2 presents example microscopic images of the aluminum alloy surface after machining. The surface roughness was measured for each layout of the adopted test plan. The example measurement results are shown in Figure 3.

**Figure 4:**
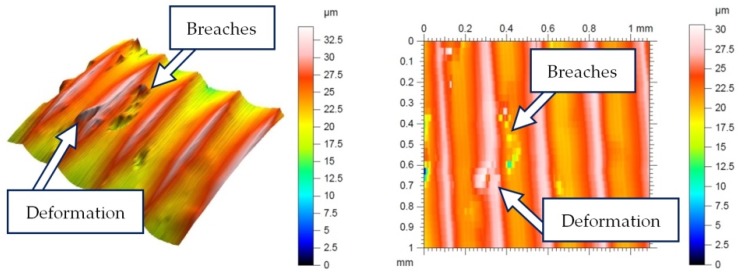
Examples of surface topography: *f* = 0.25mm/rev, *ap* = 0.5mm, *v_c_* = 300m/min, *r_ε_* = 0.8mm.

Figure 4 presents the selected topographies of the machined surface obtained from profilographical measurements. Table 5 presents the results of the measurements of the 3D parameters of surface roughness *Sv* (the maximum height of the surface pit), *Sz* (the maximum height of the surface), and *Sa* (the arithmetic mean height of the surface).

**Figure 5:**
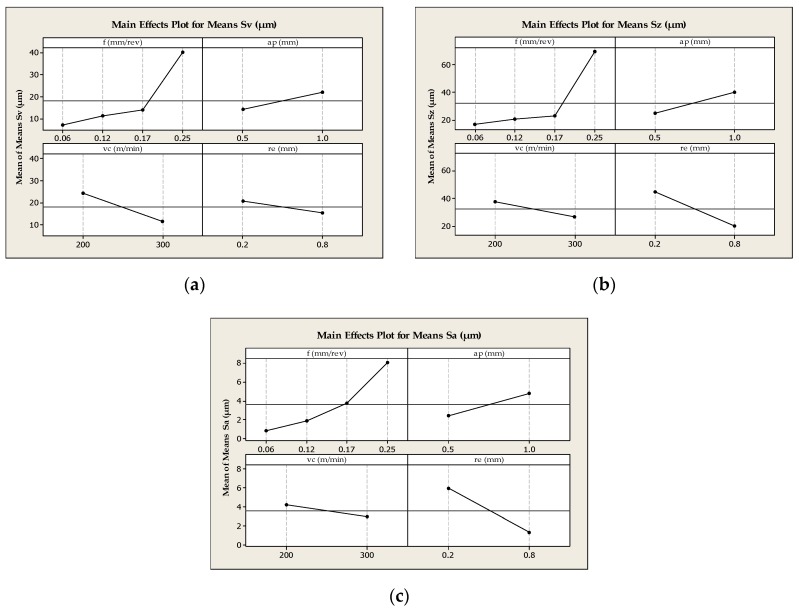
Influence of the cutting data on the values of the 3D surface parameters (**a**) *Sv* (μm), (**b**) *Sz* (μm), (**c**) *Sa* (μm).

**Figure 6:**
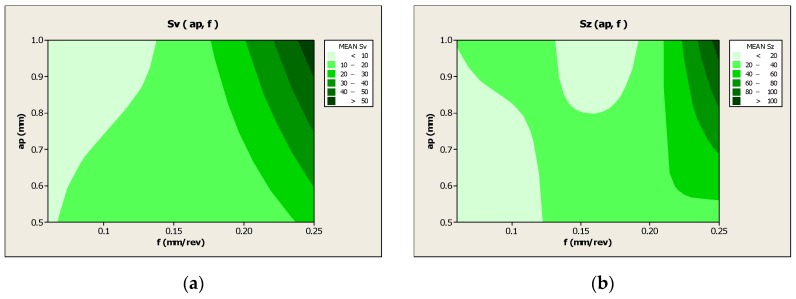
Three-dimensional surface parameters depending on the variable feed rate and depth of cut. (**a**) Parameter *Sv*(*a_p_*, *f*) (μm), and (**b**) *Sz*(*a_p_*, *f*) (μm).

Figure 5 graphically shows the influence of the particular cutting data on the values of the 3D surface parameters *Sv*, *Sz,* and *Sa*. Figure 6 shows the surface roughness parameters *Sv* and *Sz* depending on the feed variables *f* and cutting depth *a_p_*.

Table 6, Table 7 and Table 8 show the ANOVA regression analysis results of the components for the *Sv*, *Sz*, and *Sa* parameters (where: *DF*—degrees of freedom, *Seq SS*—sums of squares, *Adj SS*—adjusted sums of squares, and *Adj MS*—adjusted means squares).

Equation *Sv* (*f*, *a_p_*, *v_c,_ r_ε_*), *Sz* (*f*, *a_p_*, *v_c,_ r_ε_*) and *Sa* (*f*, *a_p_*, *v_c,_ r_ε_*) are described below (3–5).

(3) $$Sv\left(f,a_{p},v_{c},r_{\varepsilon }\right)=17.7+171f+15.2a_{p}-0.128v_{c}-9.2r_{\varepsilon },$$

(4) $$Sz\left(f,a_{p},v_{c},r_{\varepsilon }\right)=16.3+271f+30.5a_{p}-0.109v_{c}-40.8r_{\varepsilon },$$

(5) $$Sa\left(f,a_{p},v_{c},r_{\varepsilon }\right)=1.18+38.4f+4.79a_{p}-0.0121v_{c}-7.77r_{\varepsilon },$$

The analysis shows that the most important parameter influencing the values of 3D surface roughness parameters *Sv*, *Sz,* and *Sa* is the feed rate *f* and cutting depth *a_p_* (Figure 5 and Figure 6). The lowest value of the roughness pit height *Sv* was obtained for feed rate *f* = 0.06 mm/rev and depth *a_p_* = 1.0 mm. In turn, the feed rate *f* = 0.17 mm/rev and the cutting depth *a_p_* = 0.5mm at the same nose radius of cutting insert *r_ε_* = 0.8 mm results in the lowest value of the roughness height *Sz* = 10.38 μm. In addition, the analysis of the results shows that the value of the cutting speed *v_c_* and the nose radius of the cutting insert *r_ε_* have an inversely proportional influence on the 3D values of the parameters characterizing the surface roughness. Additional microscopic analyses showed a number of deformations and breaches on machined surface. The largest number of breaches were observed inside the traces (grooves) caused by cutting tool edge passage. On the other hand, numerous deformations and burrs were observed on the tops of the machining tracks appearing on the machined surface after the passage of the cutting tool. The analysis of the results shows that the number and geometric dimensions of breaches, i.e., the breach width and depth, depend on the value of cutting parameters. Surface deformations and breaches expostulate into values of 3D parameters (i.e., *Sv* and *Sz*) describing the surface roughness. The structure and properties of the subsequent layers of laser sintered material depend on the conditions of metal powder remelting, and are characterized by porosity, as mentioned by, e.g., Kempen et al. [29] and Olakanmi et al. [30] and also defects (cracks), as mentioned by Read et al. [7]. This can lead to smaller forces between the particles of the combined material and can cause the material breach out of the machined surface by the cutting tool. The dynamics of the chip-forming process and material flow direction along cutting edges in turning could affect stress distributions, as mentioned by Guo et al. [18]. In addition, the torn-out material particles can be stretched and stuck on the surface machined by the cutting tool, which deteriorates the surface roughness parameters. 

## 4. Conclusions

Based on the results obtained and the analyses carried out, the following conclusions, regarding the development of breaches formed during the turning of AlSi10MG aluminum parts made by the DMLS method, can be drawn:

(1) The values of 3D parameters describing surface roughness, i.e., *Sv*, *Sz*, and *Sa*, contain more useful information on the surface quality than 2D parameters (e.g., *Ra*, *Rz*).
(2) After lathe machining, there are numerous material breaches on the surface of sintered aluminum. The distribution of breaches and burrs is uneven. The number and dimensions of breaches, as well as material deformations and burrs on the machined surface influence the dimension and shape accuracy and performance properties of the workpieces. The depth and size of the breaches are determined by the feed rate of the cutting edge and the cutting depth. The lowest value of the 3D roughness parameters was obtained for *f* = 0.06 mm/rev, *a_p_* = 1.0 mm, and for the nose radius of the cutting insert *r_ε_* = 0.8 mm. Increasing the cutting speed value *v_c_* causes a decrease in the 3D value of the parameters *Sv*, *Sz,* and *Sa* characterizing the surface roughness. The cause of breaches and deformations on the machined surface is probably the structure of the surface layer of the sintered aluminum, and the method and conditions of combining material particles during the laser sintering process. It is likely that there are areas with weaker material particle joints that were produced by melting and subsequently by combining metal powder particles. In the absence of the full melting of the material particles during laser sintering, the cohesive forces of the particles are smaller than those of the cast material, resulting in the easier breaching (removal) of particles and plastic strain. With an increased tool feed rate in the decohesion zone, conditions are created that promote the breaching of machined material particles. An additional factor may also be the fact that there are empty spaces (pores) in sintered materials.

## Figures and Tables

**Table 1:** Properties of the powder AlSi10Mg.

Technical Data	
Recommended minimum layer thickness	30 μm
Accuracy for small items	+/− 20–50 μm
Accuracy for large items	+/− 0.2%

**Table 2:** Mechanical properties of the material AlSi10Mg.

Material	Tensile Strength Rm (MPa)	Elongation A5 (%)	Brinnel Hardness HB	Density (Sintered Part) (g/mm^3^)
AlSi10Mg	193	2.5	68	0.064

**Table 3:** Chemical composition of aluminum AlSi10Mg (%).

**Si**	**Fe**	**Cu**	**Mn**	**Mg**	**Cr**	**Ni**	**Zn**	**Ti**	**Be**	**Ca**
9.738	0.312	0.011	0.436	0.202	0.0043	<0.0020	0.0096	0.0041	<0.0003	0.006
**Cd**	**Co**	**Ga**	**Na**	**Pb**	**Sn**	**Sr**	**V**	**Zr**	**Al**	
<0.0005	<0.001	<0.002	0.001	<0.005	<0.005	<0.001	<0.002	<0.002	89.26	

**Table 4:** Research plan with real values.

No.	A	B	C	D	*f*	*v_c_*	*a_p_*	*r_ε_*
					(mm/rev)	(m/min)	(mm)	(mm)
1	1	1	1	1	0.06	200	0.5	0.2
2	1	2	2	2	0.06	300	1.0	0.8
3	2	1	1	2	0.12	200	0.5	0.8
4	2	2	2	1	0.12	300	1.0	0.2
5	3	1	2	1	0.17	200	1.0	0.2
6	3	2	1	2	0.17	300	0.5	0.8
7	4	1	2	2	0.25	200	1.0	0.8
8	4	2	1	1	0.25	300	0.5	0.2

**Table 5:** Test results for the 3D roughness parameter measurements *Sv*, *Sz,* and *Sa* (μm).

No.	A	B	C	D	*f* (mm/rev)	*v_c_* (mm/min)	*a_p_* (mm)	*r_ε_* (mm)	*S/N Sv*	*Sv__mean_* (μm)	*S/N Sz*	*Sz__mean_* (μm)	*S/N Sa*	*Sa__mean_* (μm)
1.	1	1	1	1	0.06	200	0.5	0.2	−18.8	8.73	−22.5	13.25	2.6	0.74
2.	1	2	2	2	0.06	300	1.0	0.8	−14.5	5.31	−26.1	20.15	0.8	0.91
3.	2	1	1	2	0.12	200	0.5	0.8	−23.4	14.71	−25.8	19.51	2.1	0.78
4.	2	2	2	1	0.12	300	1.0	0.2	−17.6	7.60	−26.6	21.39	−9.5	2.97
5.	3	1	2	1	0.17	200	1.0	0.2	−20.3	10.32	−31.1	35.67	−15.7	6.08
6.	3	2	1	2	0.17	300	0.5	0.8	−25.0	17.84	−20.4	10.38	−2.7	1.36
7.	4	1	2	2	0.25	200	1.0	0.8	−27.4	23.45	−29.7	30.43	−6.3	2.05
8.	4	2	1	1	0.25	300	0.5	0.2	−35.1	56.79	−40.7	107.98	−22.9	13.98

**Table 6:** Analysis of variance for means *Sv.*

Source	DF	Seq SS	Adj SS	Adj MS	F	P
A	3	1343.6	1343.6	447.9	4.11	0.344
B	1	115.0	115.0	115.0	1.06	0.491
C	1	329.9	329.9	329.9	3.03	0.332
D	1	61.3	61.3	61.3	0.56	0.590
Residual Error	1	108.9	108.9	108.9		
Total	7	1958.7				

**Table 7:** Analysis of variance for means *Sz.*

Source	DF	Seq SS	Adj SS	Adj MS	F	P
A	3	3662.9	3662.9	1221.0	0.84	0.645
B	1	465.8	465.8	465.8	0.32	0.672
C	1	236.2	236.2	236.2	0.16	0.756
D	1	1195.9	1195.9	1195.9	0.82	0.531
Residual Error	1	1454.4	1454.4	1454.4		
Total	7	7015.2				

**Table 8:** Analysis of variance for means *Sa.*

Source	DF	Seq SS	Adj SS	Adj MS	F	P
A	3	60.4	60.4	20.1	0.75	0.668
B	1	11.5	11.5	11.5	0.43	0.631
C	1	2.9	2.9	2.9	0.11	0.796
D	1	43.5	43.5	43.5	1.63	0.423
Residual Error	1	26.8	26.8	26.8		
Total	7	145.1				
